# Alveolar Ridge Preservation with a Novel Cross-Linked Collagen Sponge: Histological Findings from a Case Report

**DOI:** 10.3390/jcm12247599

**Published:** 2023-12-10

**Authors:** Roberto Abundo, Claudia Paola Bruna Dellavia, Elena Canciani, Monica Daniele, Mario Dioguardi, Marta Zambelli, Michele Perelli, Filiberto Mastrangelo

**Affiliations:** 1 Independent Researcher, 10133 Turin, Italy; 2 Department of Biomedical, Surgical and Dental Sciences, University of Milan, 20126 Milan, Italy; claudia.dellavia@unimi.it; 3 Microscopic Anatomy, University of Milan, 20126 Milan, Italy; elena.canciani@unimi.it; 4 Department of Clinical and Experimental Medicine, University of Foggia, 71122 Foggia, Italy; monica_daniele.565003@unif.it (M.D.); mario.dioguardi@unifg.it (M.D.); 5 Independent Researcher, 10133 Turin, Italy; martaz11@hotmail.com; 6 Independent Researcher, 10122 Turin, Italy; micperelli@yahoo.it

**Keywords:** alveolar ridge augmentation, bone regeneration, biomaterials, cross-linked collagen

## Abstract

Alveolar ridge preservation (ARP) is a well-documented procedure to maintain bone volume after tooth extraction in order to place implants. However, at the end of the healing process, the residual biomaterial that is not reabsorbed remains embedded in the bone over time. Ribose cross-linked biomaterials demonstrated their ability to promote osteoconduction and complete resorption. The aim of this study was to evaluate the histological healing pattern of a novel ribose cross-linked collagen sponge used as a grafting material left exposed in human sockets at the time of tooth extraction. On a single patient, non-restorable lower first molars were extracted on both sides, and a ribose cross-linked collagen sponge was placed bilaterally in the cavities and left uncovered at the end of the surgery. After six months, core biopsies were taken immediately prior to implant placement; after the sample preparation, a histological analysis was performed. The results are very promising for substitution with newly formed bone and the amount of residual material. Ribose cross-linked collagen sponge could represent a valid alternative to conventional biomaterials for ARP procedures with no need for flap advancement and/or the addition of a membrane to cover the graft, reducing the invasiveness, complexity, and costs of the treatment.

## 1. Introduction

After tooth extraction, dimensional alterations occur. The osteoclastic activity results in resorption of the crestal region of both the buccal and the lingual bone wall. Studies conducted by Araujo and Lindhe showed that alterations of the height of the bone crest at 8 weeks of healing were 1.9 ± 0.2 mm [1]. Moreover, a reduction in the height of the walls was more pronounced at the buccal than the lingual aspect of the extraction socket [1]. A 50% reduction in the buccolingual width of the bone has been estimated, in addition to a decrease in bone height at 12 months after extraction [2].

Alveolar ridge preservation (ARP) is a surgical strategy of grafting at the time of tooth extraction that enables maintenance of most of the original volume of the bone crest, with the graft undergoing major resorption when left to heal spontaneously [1,2]. ARP is indicated to prevent extensive alveolar ridge resorption after complete or partial tooth extraction, either as part of immediate implant placement interventions or to reduce the need for ancillary ridge augmentation prior to or at the time of delayed implant placement [3]. The aim of ARP is to maintain horizontal and vertical alveolar ridge forms using bone grafts: autografts, allografts, xenografts, or alloplastic materials; soft-tissue grafts; guided bone regeneration (GBR) with resorbable or non-resorbable barriers; and biologically active materials or combinations to reduce the loss of coronal alveolar bone height and width [4,5].

Following on from pioneer publications [6,7], the ability of the technique to minimize the naturally occurring resorption due to the loss of the bundle bone [8] was demonstrated in several randomized clinical trials [9,10,11,12,13,14,15,16] and has become a standard of care when extracting a tooth prior to replacement with an implant, both in the esthetic zone and in posterior areas where it is able to minimize the need for further major bone augmentation procedures.

Almost non-resorbable bone substitutes are the most documented biomaterials for such a purpose, being able to stabilize blood clots and maintain space for bone regeneration for a long time [9,10,13,15,16].

However, the presence of non-resorbed particles, potentially acting as weak spots in bone tissue over time, is an issue when ideal regeneration from a biologic point of view is expected [17].

Therefore, totally and slowly resorbable biomaterials should represent the ideal graft for natural bone regeneration, with no exogenous remnants at the end of the healing process [17,18], as long as volume maintenance over time is sufficient.

Furthermore, most published procedures report a need for covering with a barrier membrane in order to achieve optimal results, but the addition of such a barrier means an increase in both costs (directly due to the economic cost of the device and indirectly due to the longer time needed for the intervention) and surgical difficulties (due to the need for stabilization of both the membrane and the graft).

Resorbable materials have a disadvantage of an unpredictable degree of resorption, which can significantly alter the amount of bone formation [19,20]. If they are resorbed too fast, the consequential lack of rigidity means that additional support is required [20,21,22]. They also have shortcomings when trying to protect large particulate grafts [20,23]. When the membranes are exposed and/or associated with inflammatory reactions in the adjacent tissue, the enzymatic activity of macrophages and neutrophils causes the membrane to rapidly degrade, thereby affecting the structural integrity of the membrane and causing decreased barrier function and less bone regeneration or bone fill; this is particularly problematic when grafting in conjunction with implant placement, as the implant becomes unstable [20,24].

Ribose sugar is a non-toxic mediator, allowing an unlimited degree of cross-linking and providing a collagen barrier that is extremely resistant to enzymatic degradation [25]. Ribose cross-linked collagen showed long-lasting [26,27,28] and osteoconductive [29,30,31] properties, irrespective to exposure in the oral cavity [25,32,33,34], allowing bone regeneration as it is naturally resorbed. Widely documented as a suitable membrane [35,36,37,38], such a cross-linked material is also used as a multilayer matrix [39] and has been recently fabricated as a sponge, acting as a tridimensional scaffold. Cross-linked collagen membranes retain integrity for a longer period than alternatives, thus allowing better protection of the bone-healing process. While cross-linking mediated by glutaraldehyde could be a possible reason for decreased biocompatibility, cross-linking mediated by ribose sugar shows no adverse tissue reaction and retains integrity in vivo for a longer period than alternatives. Moreover, cross-linked membranes have the capacity to withstand bacterial collagenolytic degradation while facilitating soft-tissue healing over the exposed membranes [25].

A study conducted by Tal et al. [25] shows how non-cross-linked membranes can retain membrane integrity with mild peripheral resorption and membrane collagen disintegration.

The aim of the present study is to evaluate the histological healing pattern of a novel ribose cross-linked collagen sponge used as a grafting material left exposed in human sockets at the time of tooth extraction.

## 2. Materials and Methods

**Figure 1:**
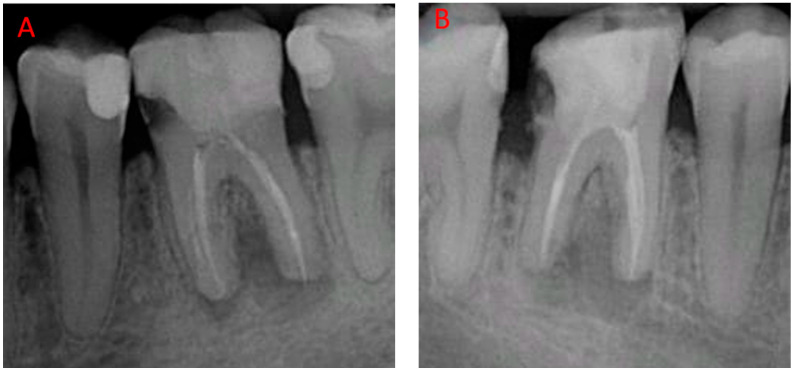
Initial periapical radiographs: (**A**) 3.6 and (**B**) 4.6. Both teeth show poor prognosis due to large periapical lesions from endodontic failures.

The patient was a 28-year-old male in good general health. Oral evaluation had shown poor prognosis for molars 3.6 and 4.6, due to a combination of unfavorable endodontic and restorative factors, with large periapical radiolucency and deep carious lesions detectable in periapical X-rays (Figure 1).

Informed consent was obtained from the patient before he participated in the study, and the case was conducted in accordance with the Declaration of Helsinki.

### 2.1. Surgical Protocol

After inferior alveolar nerve-block anesthesia with articaine plus 1:100,000 epinephrine (Alfacaina, Dentsply Sirona, York, PA, USA), supracrestal connective tissue fibers were interrupted by means of a periotome, and then, the roots were separated with a carbide multiblade bur mounted on a high-speed handpiece.

The roots were separately extracted with a lever and a forceps; after a thorough debridement of the socket with an alveolar curette, the residual cavity was rinsed with a rifamycin 250 mg/3 mL antibiotic solution (Rifocin, Sanofi, Paris, France).

A ribose cross-linked collagen sponge (Ossix^®^ Bone, Datum Dental, Lod, Israel) was trimmed starting from a 5 × 10 × 10 mm block, placed in the most coronal portion of the socket for the time necessary to become completely soaked with blood, and then inserted into the post-extraction cavity, leaving the coronal side of the sponge exposed at the same level as the gingival margin.

**Figure 2:**
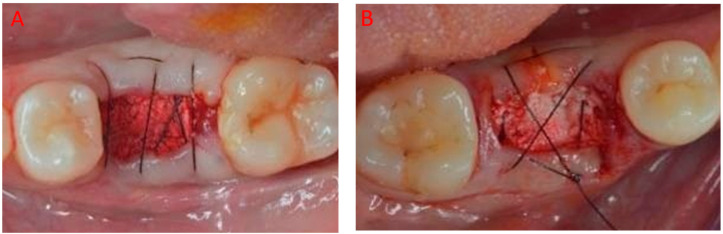
Cross-linked collagen sponge (Ossix^®^ Bone, Datum Dental, Lod, Israel) filling the socket: (**A**) 3.6 and (**B**) 4.6.

A 5.0 synthetic monofilament (Supramid, Serag-Wiessner, Naila, Germany) single sling suture crossing the wound was used to secure the sponge in place (Figure 2). The biomaterial was left intentionally uncovered, maintained in place with sutures.

According to the 2021 Italian Academy of Osseointegration (IAO) Consensus Meeting Report on antibiotic and antiseptic use in implant surgery, antibiotic (Augmentin, GlaxoSmithKline, Brentford, UK) and anti-inflammatory (Brufen, Abbott, Chicago, IL, USA) therapy and mouthrinses (Corsodyl 0.2%, GlaxoSmithKline, Brentford, UK) were administered after surgical procedures only for six days [40]. No provisional restoration was delivered prior to the expected implant placement.

After 2 weeks, the sutures were removed. The patient received another clinical control at 3 months. At 6 months, periapical X-rays and cone beam computed tomography (CBCT) were taken, and surgical reentry for implant placement was performed in a ridge clinically showing only minimal reduction in its horizontal dimension. It was planned to place implants of 5 mm diameter in the middle of each edentulous area with no surgical guide; both sites had 2 mm or more of available bone thickness on each side (buccal and lingual) of the two implants.

During surgery, after flap elevation, a trephine bur with an inner diameter of 3.0 mm was used as a pilot drill, allowing harvesting of a bone core from the middle of each edentulous site; biopsies were immediately fixed in 10% buffered formalin. After drilling with three cylindrical burs of increasing diameter (the conventional sequence according to the implant protocol), 5.0 × 8.5 mm implants (Premium, Sweden & Martina, Due Carrare, Italy) were placed and the biopsies underwent histological analysis according to the sequence below.

### 2.2. Sample Preparation

The specimens were processed for ground sections at the Laboratory of Thin Sections of the Department of Biomedical, Surgical and Dental Sciences at the University of Milan, Italy. In relation to the quantity and characteristics of the residual biomaterial, the abrasion processing of bone biopsies typically takes 30–40 days. Samples, stored in 10% formalin, are delivered within 24–48 h of collection. On arrival at the laboratory, samples undergo buffering, followed by a series of steps involving the use of alcohols and resins, leading to sample infiltration and embedding. The biopsies were washed in PBS (phosphate-buffered saline 1%) and then dehydrated in an increasing alcohol scale (70%, 80%, 90%, 96%, and 100%). Subsequently, the samples were infiltrated with a blend of methyl methacrylate resin/alcohol for inclusion in pure resin (Technovit 7200 VLC, Exact Kultzer, Bio-Optica, Milan, Italy) by light polymerization. These steps typically require an average of 24 h to 3 days. After embedding, the process proceeds to cutting by abrasion, attaching the section, and polishing. Samples were cut by a cutting machine (Micromet, Tecnocontrol, Bologna, Italy). Two sections were glued with resin (Technovit 7210 VLC, Exact Kultzer, Bio-Optica, Milan, Italy) on a plastic slide (Exact Kultzer, Bio-Optica, Milan, Italy), and ground using a grinding machine (LS2, Remet, Bologna, Italy) until about 100 µm thick. All sections were polished and, at the end of the process, stained with toluidine blue and pyronine yellow. One day was required for staining. Observation with photographic documentation and histomorphometry had a variable duration depending on the parameters analyzed and the characteristics of the sample itself.

### 2.3. Whole Slide Image and Morphological Analysis

Each slide was digitized using a high-resolution digital imaging scanner (NanoZoomer S360 digital slide scanner, Hamamatsu, Japan) at 400× to observe morphological features. Digital files were managed by Ndp.view software to assess the following parameters: (1) signs of cell-type response, to indicate necrosis and inflammatory infiltrate presence; (2) features of tissue response related to fibrosis and fatty infiltrate associated with phlogosis towards the graft; (3) presence of residual biomaterial; (4) new bone formation and bone remodeling in the grafted area; (5) biomaterial ossification processes; and (6) vascularization.

### 2.4. Histomorphometry

A digital counting grid was applied on each acquired section in order to evaluate tissue volume fraction in the grafted area. In brief, an accurate count of the test points within the various types of tissues was conducted to obtain stereological quantification and then summarized for tissue, and finally, the ratio between tissutal points and total points was calculated and expressed as a percentage. The parameters evaluated were mature ossified matrix, ossifying matrix, and medullary spaces.

## 3. Results

Healing at both sites was uneventful after ARP. At two weeks, when sutures were removed, the wound was almost completely closed where the collagen sponge was left exposed in the oral cavity at the end of the surgical procedure.

**Figure 3:**
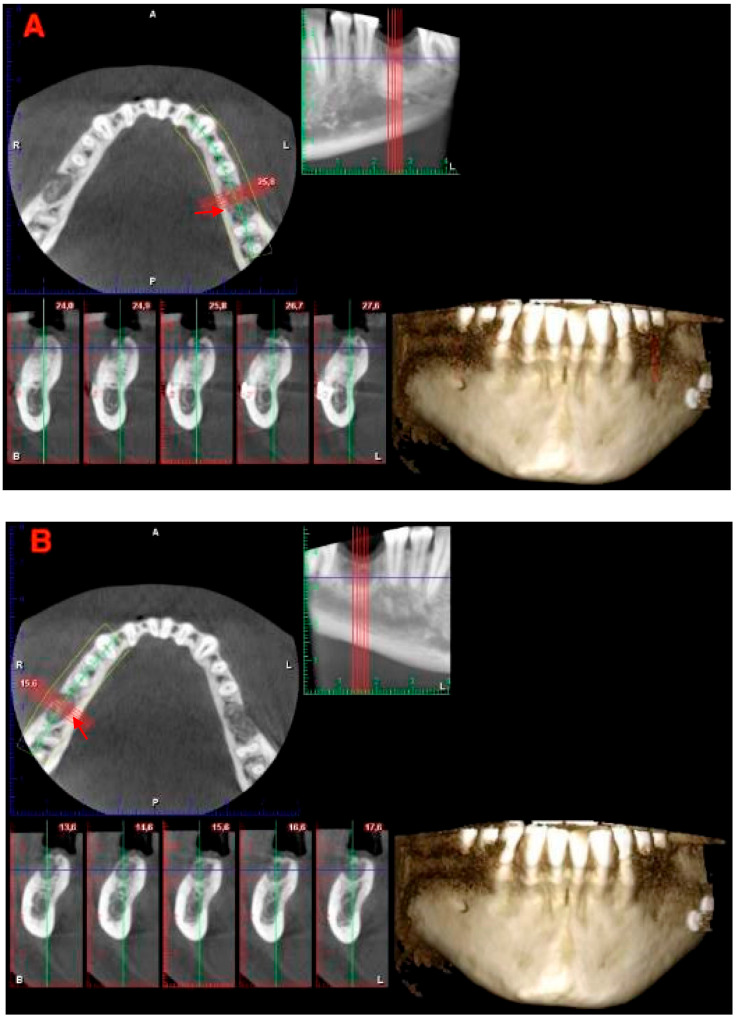
(**A**) CBCT at 6 months, 3.6. (**B**) CBCT at 6 months, 4.6. A good profile of the alveolar ridges seems to be maintained. Red arrows point the profile of the alveolar ridge.

**Figure 4:**
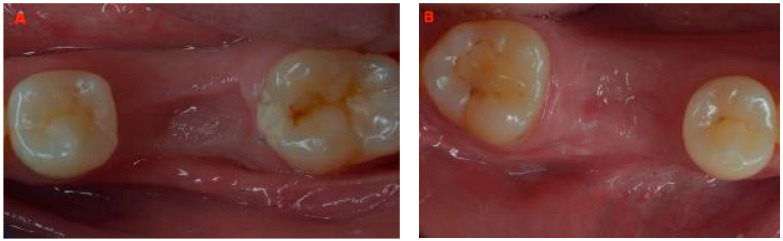
Pre-operative clinical view at 6 months: (**A**) 3.6 and (**B**) 4.6.

At six months, periapical X-rays showed good healing and mineralization, and CBCT (Figure 3) confirmed a nice ridge profile, with advanced cortical mineralization and a slight transparency in the inner portion of the newly formed bone; clinical examination found keratinized tissue covering the edentulous sites (Figure 4).

**Figure 5:**
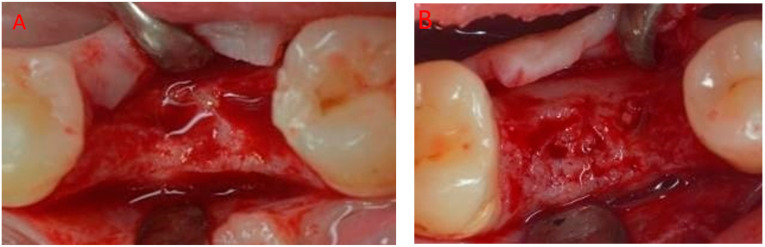
Intra-operative view after flap elevation: (**A**) 3.6 and (**B**) 4.6. A well-maintained ridge profile can be observed, with minimum reduction in the horizontal dimension; regenerated tissue and native bone are combined.

The area is covered by a good amount of keratinized tissue, 13 mm visible in Figure 5A (3.6) and 12 mm in Figure 5B (4.6).

**Figure 6:**
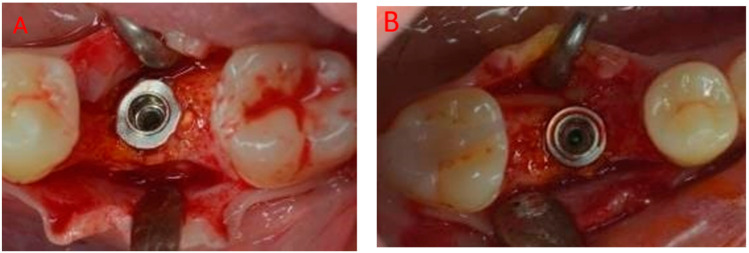
Intra-operative view after implant placement: (**A**) 3.6 and (**B**) 4.6. A 5.0 × 8.5 mm implant (Premium, Sweden & Martina, Due Carrare, Italy) was placed in both sites.

After flap elevation, a regular bone profile with a well-maintained volume was observed (Figure 5); bone density at drilling was compared with types 2–3 [41]. Implants were then placed, surrounded by a nice amount of bone (Figure 6).

### 3.1. Qualitative Evaluation of the Histological Specimens

**Figure 7:**
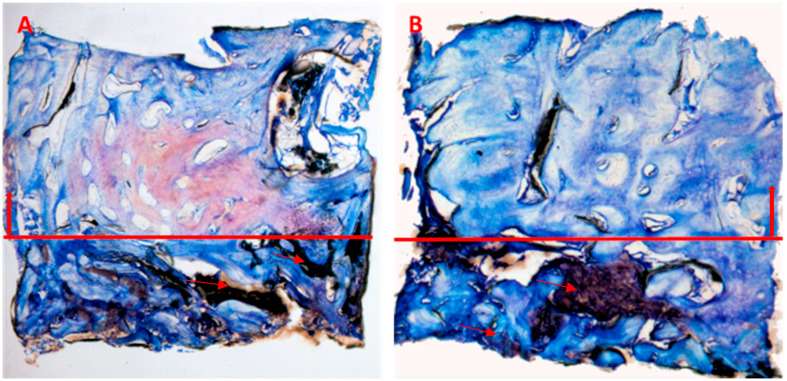
Overview: (**A**) 3.6 and (**B**) 4.6. Photos show an overview of the biopsies characterized by more mature bone structure in the coronal portion (above the red line) and immature bone structure in the apical portion (under the red line). Few residual remnants of ribose cross-linked collagen are still present (red arrows). (**A**,**B**) Total magnification 40×, toluidine blue and pyronin yellow.

The biomaterial appeared to be biocompatible, with no signs of adverse reaction from cells or tissues. No fibrosis, inflammatory infiltrates, or necrotic areas were observed. At low magnification (Figure 7), bone biopsies showed dense new bone, in particular in the coronal portion compared with the apical portion, which was characterized by a more immature pattern of mineralization.

**Figure 8:**
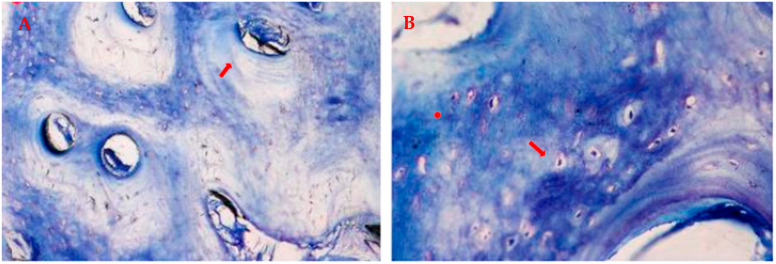
Osteons: (**A**) 3.6 and (**B**) 4.6. Photo A shows newly formed osteons immersed in ossified matrix. Lamellae are populated by osteocytes as observable in photo (**B**). (**A**) total magnification 200×, (**B**) 400×, toluidine blue and pyronin yellow. The red arrows point the newly formed osteons immersed in ossified matrix and the lamellae populated by osteocytes.

**Figure 9:**
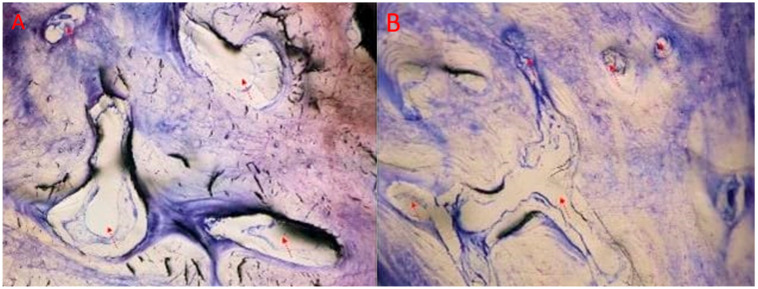
Vascularization: (**A**) 3.6 and (**B**) 4.6. Photos show numerous blood vessels characterized by thin endothelial walls hosted into neurovascular canals of the compact bone. (**A**,**B**) Total magnification 200×, toluidine blue and pyronin yellow. The arrow point the blood vessels in the compact bone.

In the coronal portion, ossified matrix was characterized by newly formed osteons, with concentric lamellae surrounding the neurovascular canal and populated by numerous bony lacunae filled with osteocytes (Figure 8). Active metabolism of the bone was indicated by Haversian canals with vessels of small and medium diameters (Figure 9).

**Figure 10:**
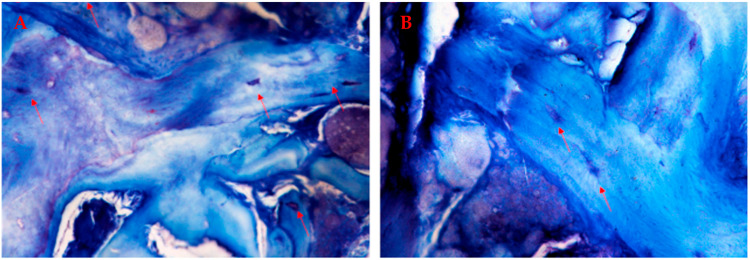
Ossifying process in the apical portion: (**A**) 3.6 and (**B**) 4.6. Photos show numerous areas of new bone matrix in close contact with ossifying matrix, recognizable by the presence of osteocytes in bone lacunae (red arrows). (**A**) Total magnification 200×, (**B**) 400×, toluidine blue and pyronin yellow.

In the apical portion, a dense osteoid matrix, rich with collagen fibers, showed that tissue strongly shifted towards the anabolic phase of mineralization. Immature matrix seemed to integrate the graft that was undergoing an ossifying process. The bone cells stained well, and activated osteocytes and numerous osteoblasts were seen in the active phase of osteoid matrix deposition in which they remained entrapped, forming a bony bridge between ossified matrix blocks (Figure 10).

No osteoclastic cells were detected, indicating that the graft provided support for the formation of a new matrix and the apposition of new mineralized tissue, promoting an anabolic bone phase without the need to resorb the material.

### 3.2. Histomorphometric Analysis

The newly formed tissue in both sites was composed of 28.75% ± 1.30% ossified matrix with a high level of mineralization, 56.42% ± 2.32% ossifying matrix in mineralization phase, and 14.83% ± 3.62% marrow space.

## 4. Discussion

After tooth extraction, maxillary pathologies or trauma, structural and compositional hard- and soft-tissue changes, as well as morphological alterations can be expected [42]. Several studies have demonstrated the effectiveness of ARP procedures in obtaining adequate bone volume for esthetic rehabilitation [10,11,13] and prosthetically driven implant placement [4]. To achieve adequate new bone formation and volume maintenance, surgical advancing of the buccal flap coronally or sealing the socket with a membrane was considered mandatory to promote primary closure of the healing site [15,16,43]. In addition, several autologous or heterologous biomaterials have been proposed to prevent loss and collapse during bone remodeling [44,45].

In 2019, G. Avila-Ortiz et al. showed that ARP via socket grafting (ARP-SG) with the application of particulate allogenic or xenogeneic materials covered with an absorbable collagen membrane or a rapidly absorbable collagen sponge was associated with the most favorable outcomes in terms of horizontal ridge preservation (buccal bone thickness > 1.0 mm) and more favorable ridge preservation value (difference between ARP (AG + SS) and control = 3.2 mm) compared with tooth extraction alone or with sites with a thinner buccal wall (difference between ARP (AG + SS) and control = 1.29 mm) [45]. In 2017, N. MacBeth et al., in a systematic review, showed that the standardized mean difference (SMD) in vertical mid-buccal bone height between ARP and a non-treated site was 0.739 mm (95% CI: 0.332 to 1.147); histological analysis after ARP indicated unclear results in the width of the keratinized tissue after GBR, with extensive variations in total bone formation (47.9 ± 9.1% to 24.67 ± 15.92%) related to the different treatment protocols and biomaterials used, and post-operative complications with soft-tissue inflammation and infection signs [46]. In 2020, E. Minetti et al., in a histological and histomorphometric analysis, showed new bone volume of around 36–39% and vital bone increases of around 20–22% in ARP sites using tooth as a graft material, with no inflammation signs in any specimens analyzed [47]. In 2020, G. Avila-Ortiz et al., in a randomized controlled trial using a combination of socket grafting with particulate bone allograft and socket sealing with a nonabsorbable membrane (dPTFE), indicated ARP as a treatment to attenuate the physiologic resorptive events that occur as a consequence of tooth extraction, for maintenance of alveolar bone and to reduce the estimated need for additional bone augmentation at the time of implant placement, and showed volumetric bone resorption significantly higher in the control group (mean ± SD: EXT = −15.83% ± 4.48%, ARP = −8.36% ± 3.81%, *p* < 0.0001). No significant differences in terms of soft-tissue contour change were observed between groups [3]. In 2022, E. Minetti at al., in a histomorphometric analysis of biopsies from 483 dental implants placed in maxillary sites after ASP procedures using demineralized tooth extracted as graft material, showed a high percentage of bone volume (BV) 43.58% (±12.09) and vital new bone (NB) 32.38% (±17.15) with an absence of inflammation or necrotic areas and a 98.2% implant-survival rate (10 implant failures) 12 months after loading [48].

The use of a novel ribose cross-linked collagen sponge for ARP showed very promising results in the present study. Combining histological with clinical outcomes, it can be observed that the material was well tolerated by the surrounding tissues and optimally acted as a scaffold despite being left exposed at the end of the first surgical procedure. This ability to allow nice keratinized tissue formation over the material to resorb and undergo ossification is consistent with previously reported data from other cases in which ribose cross-linked collagen was used in the form of a membrane [25,29,30,31,32,33,34].

In these two pilot cases, a post-operative antibiotic regimen was preferred instead of a single preoperative dose, in accordance with guidelines for complex procedures reported by the Consensus Report of the Italian Academy of Osseointegration [40], due to the direct exposure of the sponge to the oral cavity at the end of the procedure. A provisional restoration during the expected time before placing the implants was not used. In the posterior area, although the temporary restoration could contribute to space maintenance, there are indeed several concerns related to the absence of esthetic relevance, to irreversible invasiveness for the adjacent teeth with a conventional fixed partial denture, to difficult clinical management of debonding/rebonding with a resin-bonded fixed partial denture, to risk of incomplete ossification of the coronal portion of the socket with a removable partial denture, and to additional costs.

A 5 mm diameter implant was placed on each side. Such a wide-diameter fixture was planned according to the final dimension of the healed ridge, allowing a positioning with at least 2 mm of residual bone thickness both on the buccal and on the lingual side, thus creating an optimal condition for proper maintenance of peri-implant bone with time along with ideal support for a prosthetic crown in the molar region.

In this pilot case, free-hand implant placement was performed. In further studies, it would be important to use guided surgery procedures that allow more accurate implant positioning, but these would come with additional costs that always need to be evaluated in a ratio with benefits in clinical practice. The results obtained seem particularly encouraging for the quantity and quality of the regenerated bone tissue, without the presence of inflammatory tissue or adverse reactions to the surgical procedure.

The absence of intra- or post-operative complications validates the value of surgical treatment of post-extraction sites.

However, further studies will be necessary to confirm the encouraging results obtained, thanks to an increase in the number of surgical sites analyzed and bone biopsies obtained, in order to also carry out a statistical investigation.

Also, studies will be necessary to compare cross-linked collagen sponges with autologous samples or with biomaterials currently on the market and used in the same surgical procedures.

Finally, studies will be necessary that can analyze different implant placement moments to assess the best timing for implant rehabilitation [10,11,13,15,16,43,44,49]. Therefore, it should be possible to reduce invasiveness, complexity, duration, and overall costs of the surgical intervention with potential beneficial effects both for patients and clinicians. Moreover, the histological features of the newly formed tissues resemble a more natural pattern of healing. Well-organized bone was detected in biopsies; it was denser and more mature in the most coronal portion compared with the apical portion, similar to natural healing of a socket [50]. There were few residual remnants of ribose cross-linked collagen (Figure 7) in comparison with data in the literature reporting substantial amounts of non-resorbed particles of conventional biomaterials at the end of the healing process [51,52,53,54] that will not resorb over time due to their intrinsic characteristics.

## 5. Conclusions

Further investigations with a larger number of cases, a longer observation period, and randomized controlled trials comparing standard procedures will be needed to confirm the interesting results reported in the present study.

However, the histological outcomes achieved using a novel ribose cross-linked collagen sponge have shown the efficacy of the biomaterial as a positive option in ARP procedures, with healthy bone regeneration and favorable volume maintenance even when left exposed at the end of the surgical procedure.

## Data Availability

Data are contained within the article.
